# A Wearable Multi-Modal Measurement System with Self-Developed IMUs and Plantar Pressure Sensors for Real-Time Gait Recognition

**DOI:** 10.3390/mi17030371

**Published:** 2026-03-19

**Authors:** Xiuyu Li, Yunong Gao, Guanzhong Chen, Meiyan Zhang, Jingxiao Liao, Zhaoyun Wang, Jinwei Sun

**Affiliations:** 1 Electrical Measurement Technology and Intelligent Control Institute, School of Instrumentation Science and Engineering, Harbin Institute of Technology, Harbin 150080, China; 22b901047@stu.hit.edu.cn (X.L.); 25s101048@stu.hit.edu.cn (Y.G.); jwsun@hit.edu.cn (J.S.); 2 The 49th Research Institute of China Electronics Technology Group Corporation, Harbin 150028, China; shuijing2005@163.com (G.C.); wangzhaoyun4949@163.com (Z.W.); 3 Department of Data Science, City University of Hong Kong, Hong Kong, China

**Keywords:** gait recognition, wearable sensors, multi-modal fusion, IMU, plantar pressure, ensemble learning

## Abstract

To address the limitations of existing wearable gait recognition, such as drift in static actions and difficulty in recognizing transition states, this paper proposed a gait recognition system based on the data fusion of MEMS Inertial Measurement Units (IMUs) and flexible plantar pressure sensors. A low-power wearable device comprising four inertial and two pressure sensing nodes was developed to achieve synchronized multi-source data collection. Regarding the algorithm, a sensor-characteristic-based two-stage hierarchical framework was constructed. The first stage utilized plantar pressure features to efficiently decouple static postures from dynamic gaits. The second stage employed a lightweight Support Vector Machine combined with a Finite State Machine for static and transitional actions, while an ensemble learning model based on Soft Voting was used for complex dynamic gaits. Experimental results under Leave-One-Out Cross-Validation demonstrate a comprehensive recognition accuracy of 96.17%, with 100% accuracy for standing and 97% for sit-to-stand transitions. These findings validate the significant advantages of the multi-modal fusion approach in enhancing the robustness and generalization capabilities of gait recognition.

## 1. Introduction

Gait refers to the biomechanical process in which the human body achieves directional displacement in space through the coordinated movement of joints such as the hip, knee, ankle, foot, and toes. The analysis of this movement is known as gait analysis [1]. Currently, gait analysis methods can be categorized into non-wearable-based and wearable-based systems [2]. While non-wearable solutions, such as vision-based approaches and floor pressure sensors, can achieve accurate recognition [3,4], they are severely limited by viewing angles, high costs, and strict site requirements. In contrast, wearable devices demonstrate superior practicality for daily scenarios, enabling continuous, real-world monitoring for fall risk assessment, rehabilitation evaluation, and exoskeleton control [5].

For wearable devices, gait recognition methods based on Micro-Electro-Mechanical Systems (MEMS) Inertial Measurement Units (IMUs) are widely applied. Researchers have utilized smartphone or body-worn accelerometers and gyroscopes combined with Convolutional Neural Networks (CNNs) to classify daily activities [6,7]. Other approaches have employed specialized low-pass filters [8], fuzzy logic models [9], particle swarm optimization for sensor placement [10], and hip joint angle analysis [11] to capture kinematic data. Despite these advances, relying solely on inertial data faces inherent challenges. Deep learning models applied to IMU data often incur high computational costs, limiting their deployment on resource-constrained devices [7]. More critically, IMUs suffer from inherent zero-bias drift during static postures, leading to poor recognition of sitting and standing states, and they frequently confuse highly similar dynamic gaits, including stair climbing versus level walking, due to feature overlap [6,8].

Apart from inertial sensors, joint angle sensors, surface electromyography (sEMG), and plantar pressure sensors are also utilized for gait analysis [12]. For instance, integrating sEMG signals from multiple lower limb muscles can achieve high recognition rates [13]; however, weak bio-signals and uneven muscle distribution in depth [14] place excessively high demands on sensor installation precision. In contrast, plantar pressure sensors can reliably capture foot–ground contact and weight distribution [15]. Recent studies have developed smart insoles based on Support Vector Machines (SVMs) [16] and slippers equipped with flexible piezoelectric sensors [17] to extract gait parameters. Nevertheless, these modalities have critical physical limitations: piezoelectric sensors suffer from charge leakage and cannot detect constant pressure during static standing [17], while piezoresistive sensors fundamentally lack spatial kinematic information (such as foot trajectory) during the Swing Phase when the foot is off the ground.

In summary, single-modality systems struggle to provide robust continuous daily monitoring. They typically exhibit high misclassification rates during transitional movements (e.g., sit-to-stand) [18]. Furthermore, their generalization is often severely limited when deployed across different populations [19]. To address these fundamental limitations, this paper proposes a wearable gait recognition system based on the data fusion of IMUs and flexible plantar pressure sensors. By independently developing a low-power multi-modal wearable device, this system leverages the reliable weight-bearing context of pressure sensors during static states and the continuous spatial orientation of IMUs during dynamic phases. The main contributions of this study are highlighted as follows:

(1) **A Coarse-to-Fine Hierarchical Framework Based on Multi-modal Fusion:** A two-stage algorithm was constructed to efficiently decouple static/transitional states from dynamic gaits using plantar pressure features. This hierarchical routing fundamentally circumvented the zero-bias drift of IMUs in static postures and significantly reduced the overall computational burden for edge deployment.
(2) **Enhanced “Steady-Transition” Recognition for Continuous Real Gaits:** Addressing the high misrecognition probability of transitional actions in existing isolated-gait solutions, we developed a lightweight Support Vector Machine (SVM) combined with a Finite State Machine (FSM). This logic ensured the physical continuity of action states, enabling highly robust tracking of continuous real-world transitional gaits, specifically sit-to-stand and stand-to-sit.
(3) **Improved Generalization via Ensemble Learning and Biomechanical Indicators:** For complex dynamic gaits, the algorithm introduced an ensemble learning model based on a Soft Voting mechanism, fusing the strengths of a Dual-Branch Neural Network, Random Forest (RF), and Gradient Boosting Decision Trees (GBDTs). Additionally, the integration of domain-specific biomechanical indicators, such as Dual Support Period, Center of Pressure, and Range of Motion, significantly enhanced the model’s feature representation and cross-population generalization capabilities.

## 2. Materials and Methods

### 2.1. System Architecture and Hardware Design

#### 2.1.1. System Overview

**Figure 1:**
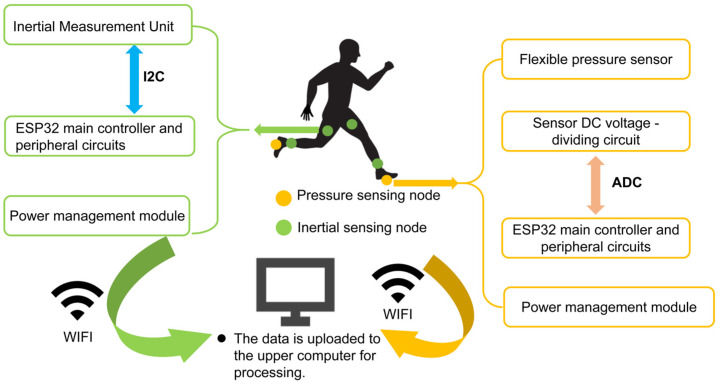
Block diagram of the multi-modal gait recognition system architecture.

The overall architecture of the proposed gait recognition system is illustrated in Figure 1. The system consists of two main components: the wearable data acquisition terminals and the host computer processing software.

The wearable terminal includes four inertial sensing nodes (placed on the thighs and shanks) and two plantar pressure sensing nodes (integrated into insoles). The main controller employs the ESP32 module (a RISC-V 32-bit single-core microprocessor, Espressif Systems, Shanghai, China), which manages data synchronization and transmission.

All nodes are powered by rechargeable lithium batteries to ensure portability. The collected data is transmitted wirelessly to the host computer via Wi-Fi for real-time processing. Compared to Bluetooth, the Wi-Fi protocol was selected for its broader bandwidth and stability in this specific application scenario.

The host computer deploys a two-stage adaptive fusion algorithm based on sensor characteristics. Usually, pressure sensor and inertial sensor data complement each other. Using sliding window segmentation, dozens of time-domain and frequency-domain features are extracted to achieve relatively precise gait classification.

#### 2.1.2. Sensor Nodes

For the pressure sensing nodes, we utilized the DF9-40 flexible piezoresistive sensors from Leanstar (Suzhou, China). We adopted an integrated sensor insole scheme.

To quantitatively evaluate the sensor’s reliability in practical applications, we analyzed its measurement range, overload capacity, and long-term durability. According to the manufacturer’s specifications, the pressure-sensitive area was a circle with a diameter of 7.5 mm, corresponding to an effective sensing area of 4.418 × 10^−5^ m^2^. Based on this area, a localized force of 100 N corresponded to a maximum measurable plantar pressure of approximately 2263.5 kPa. Established biomechanical studies on plantar pressure distribution during locomotion indicate that the peak plantar pressure of healthy adults, even during dynamic activities such as running, generally remains below 800 kPa across different foot regions [20]. Therefore, the expected localized impact load during daily dynamic activities was well below the physical measurement limit, providing a sufficient overload margin for the system.

The durability of the piezoresistive sensors was validated through endurance tests, demonstrating the capacity to withstand over 1,000,000 pressing cycles without structural failure. The sensors exhibited a repeatability error of ±5% and a hysteresis of +15%. Although continuous static loading may have induced a minor resistance drift of up to 5%, the proposed dynamic gait application inherently mitigated this effect. Specifically, during the Swing Phase, the plantar pressure dropped to zero (>10 MΩ), providing periodic mechanical unloading that prevented the accumulation of static zero drift. Furthermore, the recognition algorithm relied on periodic Time-Domain Energy thresholds rather than absolute static amplitudes, ensuring high system resilience to potential sensitivity degradation after repeated use [21].

**Figure 2:**
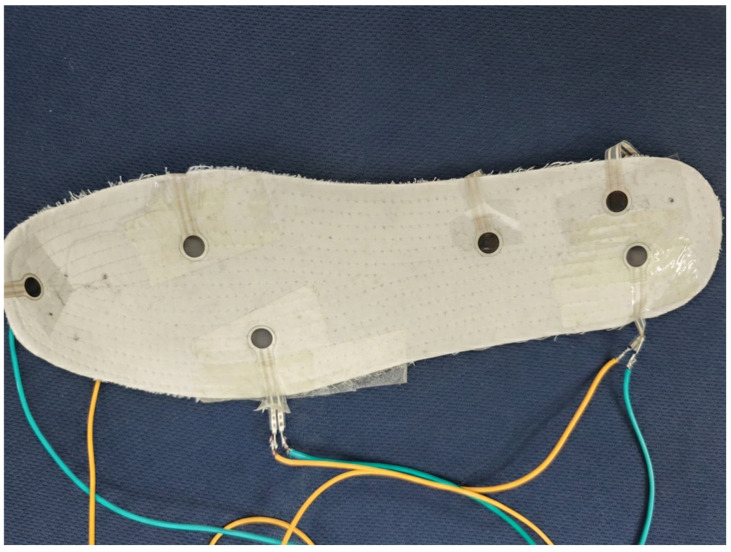
Wearable insole integrated with six flexible piezoresistive sensors.

Based on the variations in plantar pressure during human movement, we arranged six sensors on each foot, specifically positioning them at the heel, arch, and toes. The sensors were placed underneath the insole and secured with tape. The wearable insole based on plantar pressure is shown in Figure 2; the insole is placed inside the shoe and the wiring is secured to the inner lining of the shoe using tape.

**Figure 3:**
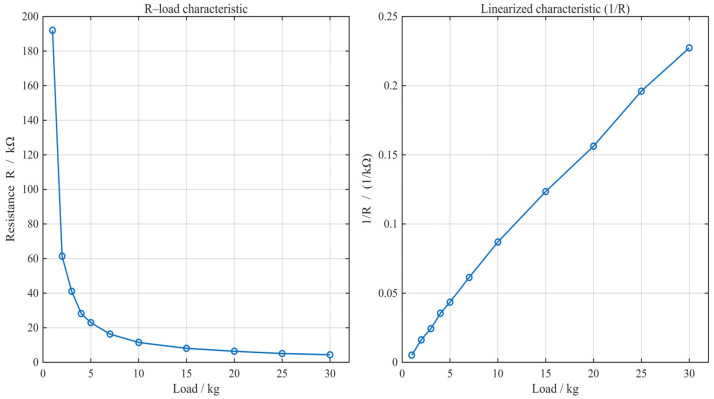
R-load characteristic and linearized characteristic.

The sensors used in this study are piezoresistive; their resistance decreases as the applied pressure increases, establishing a unique correspondence between resistance and pressure values. By referring to the manufacturer’s datasheet and conducting multiple measurements with a multimeter, we fitted the characteristic curve correlating sensor resistance to the applied load, as shown in Figure 3.

Existing methods for measuring resistance changes primarily include the DC voltage divider method, the bridge circuit method, and the operational amplifier-based method. The bridge circuit requires many components, which is unfavorable for the miniaturization of the circuit board. The operational amplifier-based method (Op-amp) increases circuit design complexity and PCB footprint. In contrast, the DC voltage divider circuit is simple and well-suited for this design.

Based on calculations, a 20 kΩ resistor was selected for the voltage divider.

For the main controller, this study employed the ESP32 module (a RISC-V 32-bit single-core microprocessor). The computer served as the host computer, while the sensing nodes functioned as Sensor Nodes, utilizing Wi-Fi for information transmission. Compared to Wi-Fi, Bluetooth transmission has a shorter range (only 30–50 m), while 4G, despite its extensive base station coverage, involves high module costs and power consumption, limiting the battery life of wearable sensing devices.

For the inertial sensing nodes, we combined the LIS3MDL (3-axis magnetometer, STMicroelectronics, Geneva, Switzerland) and the ICM-42688-P (6-axis motion tracking device, including a 3-axis gyroscope and a 3-axis accelerometer, TDK InvenSense, San Jose, CA, USA) to form a 9-axis Inertial Measurement Unit (IMU). The magnetometer was particularly crucial for correcting the accumulated Yaw drift inherent to the gyroscope.

This design employed a heterogeneous sampling rate strategy: the magnetic field changed relatively slowly, so it was set to a medium frequency to conserve power; the inertial sensors used a higher frequency to minimize latency. The synthesized sampling rate was set to 100 Hz.

### 2.2. Participants and Data Collection Protocol

#### 2.2.1. Participants and Ethical Statement

Data collection was conducted with six healthy volunteers (Subject A–F; 3 males and 3 females). All participants were free from musculoskeletal or neurological disorders that could affect gait. The cohort covered a diverse range of body types to ensure data diversity. The study was conducted in accordance with the Declaration of Helsinki.

#### 2.2.2. Experimental Setup

During the experiment, both the inertial and plantar pressure sensing nodes were active with a synchronized sampling rate of 100 Hz.

Inertial Nodes: Four IMU nodes were attached to the lower limbs using elastic straps. Specifically, they were positioned 2 cm above the knee joint (distal thigh) and 2 cm above the ankle joint (proximal shank) on both the left and right legs to capture kinematic data.

Pressure Nodes: The smart insoles were inserted into the participants’ shoes to capture plantar pressure distribution.

#### 2.2.3. Data Collection Protocol

The collected daily activities were categorized into two main groups: static/transitional actions and dynamic gaits.

**Static and Transitional Actions:** This category includes static postures (standing, sitting) and transitional movements (Sit-to-Stand, Stand-to-Sit). For static postures, data was collected continuously for 150 s per state. For transitions, subjects performed repetitive actions within a 150 s window, and valid action cycles were manually extracted.

**Dynamic Gaits:** This category includes periodic movements (walking, running, stair ascent, stair descent). Subjects performed each dynamic activity continuously for 150 s to ensure sufficient periodic cycles were captured.

To verify the model’s generalization ability across different individuals, this study adopted the Leave-One-Out Cross-Validation (LOOCV) strategy. Six rounds of experiments were conducted; in each round, data from one subject was used as the test set, while the remaining five served as the training set.

#### 2.2.4. Action Definitions

The specific definitions for the eight activities are detailed in Table 1.

### 2.3. Data Pre-Processing

#### 2.3.1. Inertial Data Kalman Filtering

For the inertial sensing nodes, the InvenSense MPL library was used to fuse the 9-axis data and solve for quaternions, which were then converted into Euler angles. The solution principle was based on Kalman Filtering.

The Kalman Filter time update equation is as follows:

(1) $$\begin{array}{l} {\hat{x}}_{k}=A{\hat{x}}_{k-1}+Bu_{k-1}\\ P_{k}=AP_{k-1}A^{T}+Q \end{array}$$

The Kalman Filter state update equation is as follows:

(2) $$\begin{array}{l} K_{k}=\frac{P_{k}^{-}H^{T}}{HP_{k}^{-}H^{T}+R}\\ {\hat{x}}_{k}={\hat{x}}_{k}^{-}+K_{k}(z_{k}-H{\hat{x}}_{k}^{-})\\ P_{k}=(I-K_{k}H)P_{k}^{-} \end{array}$$

#### 2.3.2. Correction of Gimbal Lock in Euler Angle Systems Using an Unwrapping Algorithm

During gait data collection, since the Euler angles output by the sensor are limited to the interval (−180°, +180°], a periodic jump of ±360° occurs when the actual rotation angle exceeds this range, which severely interferes with normal data analysis. To obtain a continuous motion trajectory, an unwrapping algorithm is required to process the raw angle sequence.

(3) $$\begin{array}{l} \Delta \theta_{t}=\theta_{\mathrm{raw}}(t)-\theta_{\mathrm{raw}}(t-1)\\ k_{t}=\left\{\begin{array}{ll} k_{t-1}+1, & \mathrm{if}\;\Delta \theta_{t}<-180{}^{\circ}\\ k_{t-1}-1, & \mathrm{if}\;\Delta \theta_{t}>180{}^{\circ}\\ k_{t-1}, & \mathrm{otherwise} \end{array}\right.\\ \theta_{\mathrm{corr}}(t)=\theta_{\mathrm{raw}}(t)+k_{t}\cdot 360{}^{\circ} \end{array}$$

The correction logic is shown in Equations (2) and (3), where *θ*_raw_(*t*) represents the raw Euler angle output by the sensor at time *t*, Δ*θ_t_* is the angle increment between adjacent sampling times, *k_t_* is the introduced integer cycle correction coefficient, and *θ*_corr_(*t*) is the ultimately obtained continuous Euler angle sequence.

### 2.4. Feature Extraction

#### 2.4.1. Feature Extraction from Plantar Pressure Sensors

**Figure 4:**
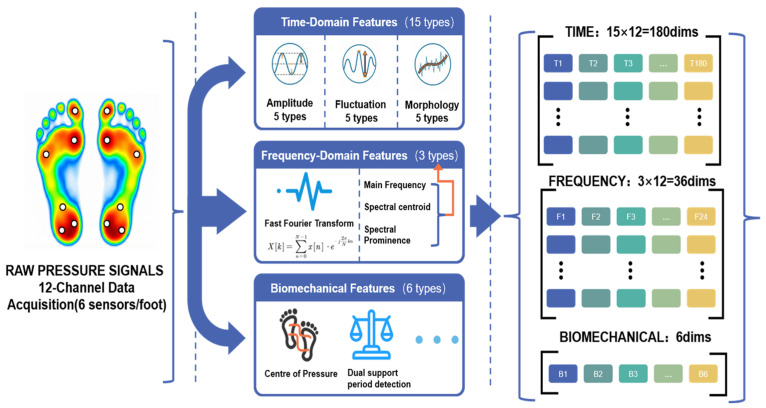
Flowchart of feature extraction and signal processing for plantar pressure data.

First, the 12-channel plantar pressure data is extracted. The overall flowchart of feature extraction and signal processing for the plantar pressure data is illustrated in Figure 4. As shown in the raw pressure signals on the left of the figure, the colors represent the typical plantar pressure distribution (where warmer colors indicate higher pressure areas), and the white dots correspond to the specific placement locations of the six flexible piezoresistive sensors on each foot (heel, arch, and toes). This paper adopts a sliding window method to segment the data. The sampling rate of the pressure sensing nodes is 100 Hz. To facilitate the subsequent Fast Fourier Transform (FFT), the window size is selected as 128 points (1.28 s) with a step size of 32 points (meaning a 75% overlap between adjacent windows). A smaller step size helps to improve temporal resolution.

The Savitzky–Golay (SG) Filter is a time-domain filtering method widely used in sensor signal processing. Its core principle is based on local polynomial least squares fitting. By performing polynomial regression analysis on the sampled data within a sliding window, it effectively suppresses high-frequency noise. The SG Filter has the following significant characteristics: (1) frequency-domain characteristics: effectively filters out high-frequency noise components in the signal; (2) time-domain shape preservation: maintains the original shape and pulse width of the signal well during denoising; and (3) least squares optimality: achieves optimal polynomial fitting within the sliding window based on the least squares criterion. This method effectively suppresses noise while preserving useful signal features.

Assuming a gait data window of length *p* is denoted as *x*(*i*), where *i* = −*m*, …, 0, …, *m* (i.e., 2*m* + 1 = *p*), we fit the raw data within this window using a *k*-th degree polynomial:

(4) $$f(i)=a_{0}+a_{1}i+a_{2}i^{2}+\cdots +a_{k}i^{k}=\sum_{n=0}^{k}a_{n}i^{n}$$

The residual of the least squares fitting is given by:

(5) $$\begin{array}{ll} \varepsilon & =\sum_{i=-m}^{m}(f(i)-x(i{))}^{2}\\ & =\sum_{i=-m}^{m}(\sum_{n=0}^{k}a_{n}i^{n}-x(i{))}^{2} \end{array}$$

To minimize the residual, the partial derivatives with respect to each polynomial coefficient must be zero:

(6) $$\partial \varepsilon \partial_{ar}=0,r=0,1,\ldots k-1,k$$

This yields:

(7) $$\frac{\partial \varepsilon }{\partial a_{r}}=\sum_{i=-m}^{m}2(f(i)-x(i)),i^{r}=0$$

where *r* = 0, 1, …, *k*−1, *k*. Through derivation, we obtain a system of linear equations for the fitting coefficients:

(8) $$A^{T}Aa=A^{T}X$$

The form of Matrix *A* is shown in Equation:

(9) $$A=\left[\begin{array}{cccc} 1 & -m & \ldots & {\left(-m\right)}^{k}\\ \vdots & \vdots & \ddots & \vdots \\ 1 & m & \ldots & m^{k} \end{array}\right]$$

Finally, the coefficients can be solved as:

(10) $$a={\left(A^{T}A\right)}^{-1}A^{T}X$$

Based on these coefficients, the smoothed result of the *k*-th degree polynomial fitting for the window’s raw data is obtained.

In the frequency domain, the signal first undergoes a Discrete Fourier Transform (DFT). Subsequently, the absolute value of the transform result is taken. To reduce the effect of spectral leakage, a Hanning window is introduced.

(11) $$X[k]=\sum_{n=0}^{N-1}x[n]\cdot e^{-j\frac{2\pi }{N}\mathrm{kn}}$$

With a sampling frequency *f_s_* = 100 Hz and *N* = 512 points, according to Equation (11):

(12) $$\Delta f=\frac{f_{s}}{N}$$

The resolution can reach 0.195 Hz, meeting the requirements of gait analysis.

The Main Frequency and Spectral Centroid can be obtained. The Main Frequency is defined as the frequency component with the largest spectral amplitude within the physiological frequency bandwidth of the human gait:

(13) $$f_{dom}=\underset{f\in [f_{\min},f_{\max}]}{\arg\max}S(f)$$

This study sets the physiological frequency bandwidth of human gait to 0.5–20 Hz to exclude the interference of DC components and high-frequency noise.

The Spectral Centroid formula is as follows:

(14) $$F_{centroid}=\frac{\sum_{f=f_{min}}^{f_{max}}f\cdot S(f)}{\sum_{f=f_{min}}^{f_{max}}S(f)}$$

After obtaining the Main Frequency, the Spectral Prominence can also be calculated:

(15) $$\begin{array}{l} X_{cop}(t)=\frac{\sum_{i=1}^{12}P_{i}(t)\cdot x_{i}}{\sum_{i=1}^{12}P_{i}(t)}\\ Y_{cop}(t)=\frac{\sum_{i=1}^{12}P_{i}(t)\cdot y_{i}}{\sum_{i=1}^{12}P_{i}(t)} \end{array}$$

Therefore, for a single pressure channel, there are 15 time-domain features and 3 frequency-domain features. With 12 pressure channels, there are 216 dimensions. Additionally, there are 6 global biomechanical features, so the total dimensionality of the plantar pressure feature vector is 222.

#### 2.4.2. Feature Extraction from IMUs

The processing of inertial information and the algorithm recognition flow are described as follows: After completing the preliminary processing of Euler angle data, feature extraction is performed. The sliding window method is used to segment the data, with a window size of 128 frames and a step size of 32 frames.

In the time domain, a total of 14 features are extracted:

- Amplitude-based: Maximum, Minimum, Mean, Root Mean Square (RMS), and Absolute Mean.
- Fluctuation-based: Variance, Standard Deviation, Peak-to-Peak, and Interquartile Range (Q3–Q1).
- Morphology-based: Skewness, Kurtosis, and zero-crossing rate.

In the frequency domain, after performing DFT on the signal, 10 features are extracted: Main Frequency, Mean Amplitude, Median Amplitude, Maximum Amplitude, Amplitude Standard Deviation, Low-band Energy Ratio, Mid-band Energy Ratio, High-band Energy Ratio, Spectral Centroid, and Spectral Bandwidth.

The formula for Amplitude Standard Deviation is as follows:

(16) $$\sigma_{A}=\sqrt{\frac{1}{N}\sum_{k=0}^{N-1}(A_{k}-\mu_{A}{)}^{2}}$$

The formula for Spectral Centroid is as follows:

(17) $$C=\frac{\sum_{k=0}^{N-1}f_{k}\cdot P_{k}}{\sum_{k=0}^{N-1}P_{k}}$$

The formula for Spectral Bandwidth is as follows:

(18) $$B=\sqrt{\frac{\sum_{k=0}^{N-1}P_{k}\cdot {\left(f_{k}-C\right)}^{2}}{\sum_{k=0}^{N-1}P_{k}}}$$

For a single inertial sensing node, (14 + 10) × 3 = 72 features are extracted for the three angles. Since there are four nodes, the total dimensionality of the feature vector is 288.

In addition, 4 Range of Motion (ROM) indicators (calculating the difference between the maximum and minimum values of the Euler angles of the four sensors within the sliding window) are introduced, along with the Cross-correlation Coefficient between the left-thigh Euler angle sequence and the right-thigh Euler angle sequence.

### 2.5. Hierarchical Recognition Framework

#### 2.5.1. Overview of the Coarse-to-Fine Strategy

**Figure 5:**
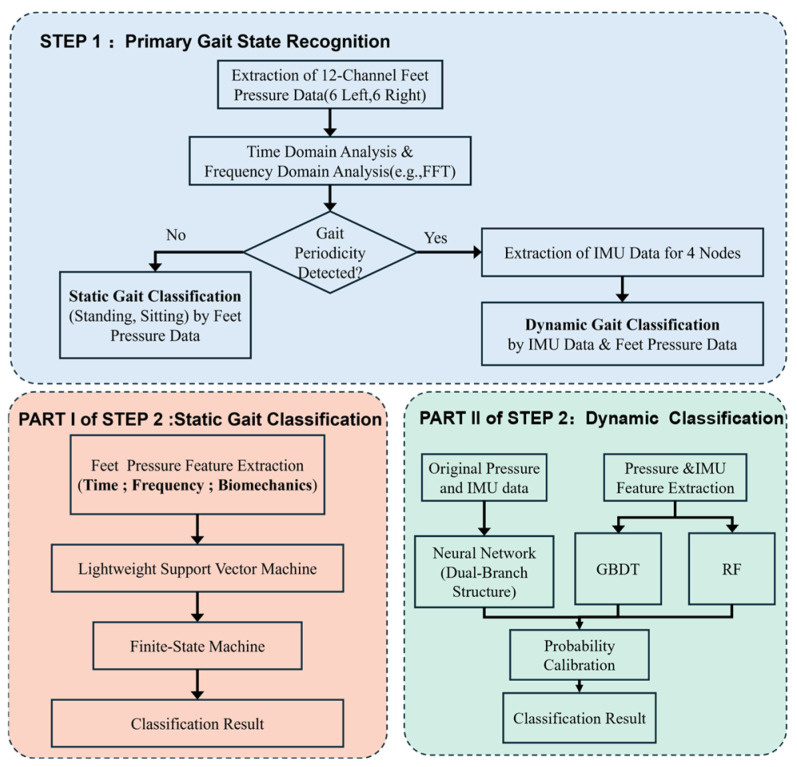
Coarse-to-fine hierarchical recognition framework.

To achieve robust and efficient gait recognition in complex scenarios, this study proposes a two-stage hierarchical recognition algorithm based on multi-modal data fusion. The specific flowchart is shown in Figure 5.

The algorithm adopts a “coarse-to-fine” processing strategy, primarily divided into two stages:

Step 1: Primary Gait State Recognition (Coarse-grained Classification). The first stage aims to distinguish between static postures and dynamic movements. We use 12-channel plantar pressure data as the primary criterion. By performing Time-Domain Analysis and Frequency-Domain Analysis (Fast Fourier Transform, FFT) on the pressure sequence, the algorithm detects whether there is dominant gait periodicity in the signal.

- If no periodicity is detected, the system determines that the subject is in a static or transitional state, activating the Static Gait Classification Branch.
- If periodicity is detected, the system determines that the subject is performing dynamic motion like walking and running, activating the Dynamic Gait Classification Branch. At this point, the system synchronously retrieves data from the 4-node Inertial Measurement Unit (IMU) for fused analysis with the pressure data.

Step 2: Advanced Pattern Classification (Fine-grained Classification)

Part I: Static Gait Classification. For static actions (e.g., standing and sitting) and transitional actions (e.g., standing up), we adopt a computationally efficient lightweight scheme. First, multi-dimensional features are extracted from the pressure data, covering time-domain statistics, frequency-domain components, and biomechanical parameters (e.g., Center of Pressure, COP). These features are input into a lightweight Support Vector Machine (SVM) for preliminary classification. To ensure the logical continuity of actions and eliminate physically impossible state mutations, a Finite State Machine (FSM) is used to post-process the SVM predictions for logical smoothing and correction.

Part II: Dynamic Gait Classification (Ensemble Learning Strategy). For complex dynamic gaits, we construct an ensemble learning model that fuses raw signal features with manually designed features:

Deep Learning Branch: Raw pressure and IMU sequences are input into a Dual-Branch Neural Network, utilizing the deep network to automatically extract high-dimensional latent dependencies from the multi-modal data.

Machine Learning Branch: Simultaneously, feature vectors containing pressure and IMU indicators are constructed and separately input into Gradient Boosting Decision Tree (GBDT) and Random Forest (RF) classifiers, leveraging their stability on structured data.

Finally, a Probability Calibration module is used to weight and fuse the output probabilities of the Neural Network, GBDT, and RF. This Soft Voting mechanism outputs the final classification result, ensuring a higher accuracy and generalization capability than any single model.

#### 2.5.2. Stage 1: Primary Gait State Recognition

**Figure 6:**
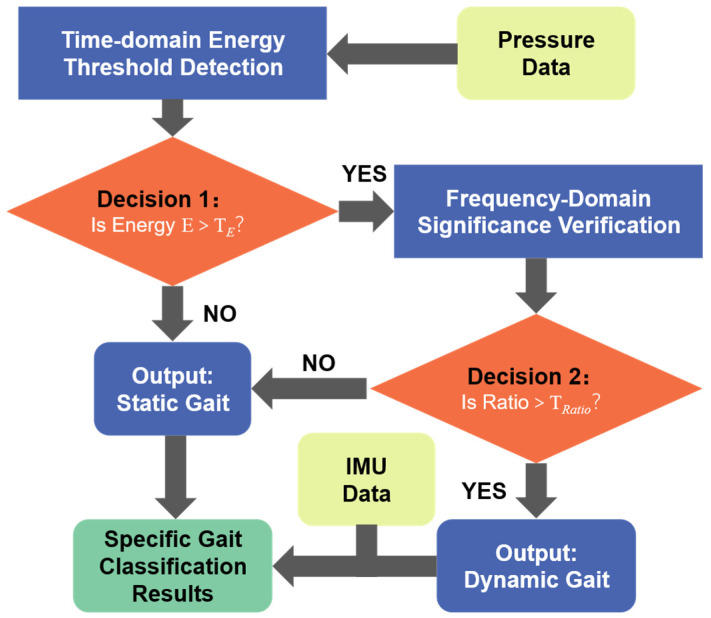
Flowchart of the primary gait state determination algorithm.

For the determination of “Dynamic Gait” and “Static Gait,” a dual-threshold mechanism combining “Frequency-Domain Significance + Time-Domain Energy Threshold” is adopted, as illustrated in Figure 6.

First, we calculate the Time-Domain Energy of the plantar pressure signal within the sliding window. A static energy threshold TE is set. If the current signal energy is lower than this threshold, it indicates that the human body is in a static or weak activity state, and the system directly determines it as “static gait”, skipping subsequent calculations.

For signals that pass the energy screening, the spectral significance (ratio), is calculated, and a Frequency-Domain Significance threshold TRatio is set. When the spectral significance is greater than the threshold, it indicates that the signal has highly concentrated periodic characteristics, and the system determines the current state as “dynamic gait”.

#### 2.5.3. Stage 2: Advanced Pattern Classification

Using only plantar pressure data, time-domain statistical features, frequency-domain features, and biomechanical features (such as COP) are extracted as input vectors. Given that the feature boundaries of static actions are relatively clear, a computationally efficient lightweight Support Vector Machine (SVM) is used for preliminary classification. To eliminate misjudgments and ensure the logical continuity of actions, a Finite State Machine (FSM) is introduced to post-process the SVM output, rejecting physically impossible state mutations, for instance, jumping directly from ‘sitting’ to ‘standing’ without a transition state, thereby achieving robust posture recognition.

In terms of the dynamic gait recognition algorithm, an ensemble model based on Soft Voting is designed. This algorithm adopts a dual-branch parallel processing architecture, combining the advantages of deep learning and traditional machine learning.

The Deep Learning Branch employs a Dual-Branch 1D Convolutional Neural Network (1D-CNN) to independently process the raw plantar pressure and IMU sequences. Each branch comprises two cascaded convolutional blocks—sequentially containing a Conv1D layer, Batch Normalization, ReLU activation, and Max Pooling—to automatically extract nonlinear temporal dependencies. The extracted feature maps are flattened via Global Average Pooling (GAP), concatenated, and fed into a fully connected (FC) layer with a Softmax output. For training, the model utilizes categorical cross-entropy loss and the Adam optimizer (learning rate = 0.001, batch size = 64). To prevent overfitting, a Dropout rate of 0.4 is applied to the FC layer, coupled with an Early Stopping mechanism (patience = 10 epochs, maximum 100 epochs).

The previously extracted pressure and IMU feature vectors are input into the machine learning branch, which uses the Support Vector Machine (SVM) and Random Forest (RF) models.

To achieve optimal performance for each model, a parameter tuning function is designed. This function employs a Grid Search strategy to try various hyperparameter combinations and determine the optimal configuration.

After the three models output their prediction results in parallel, Probability Calibration is performed first. GBDT uses Platt Scaling, Random Forest uses Voting Averaging, and the Neural Network uses Temperature Scaling.

Subsequently, the probabilities output by the three models are weighted and fused. The final ensemble probability distribution Pfinal is a weighted linear combination of the probability distributions of each base model:

(19) $$P_{final}(y=k|S,v)=\sum_{j\in \{NN,SVM,RF\}}w_{j}\cdot p_{j}^{\left(k\right)}$$

where *ω_j_* is the weight parameter assigned to each model, satisfying the normalization constraint:

(20) $$\sum w_{j}=1,\;\;w_{j}\geq 0$$

## 3. Results

### 3.1. Spatiotemporal Characteristics of Gait Signals

#### 3.1.1. Pressure Data Analysis

**Figure 7:**
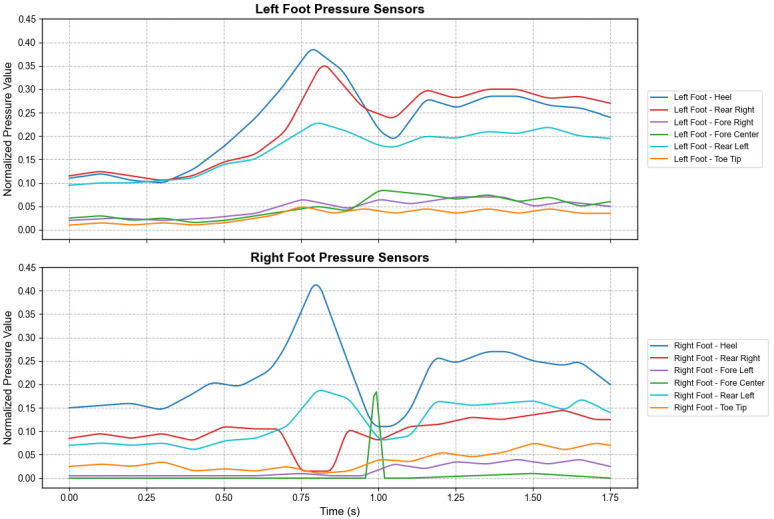
Plantar pressure variations in left and right feet during the sit-to-stand transition.

The visualization of the sit-to-stand process is shown in Figure 7. As observed, during the standing-up process, the heel pressure initially increases, then drops rapidly. Almost simultaneously, the forefoot pressure rises. After reaching a peak, the forefoot pressure decreases while the heel pressure rises again, eventually tending towards stabilization. This sequence reflects the transfer of the Center of Gravity (COG): shifting from posterior to anterior and then adjusting back from anterior to posterior to maintain balance. This demonstrates a transitional period of body stabilization from sitting to standing. Additionally, it can be observed that in the sitting posture, the pressure on both the heel and forefoot is relatively low, whereas in the standing state, the pressure is significantly higher, particularly at the heel.

**Figure 8:**
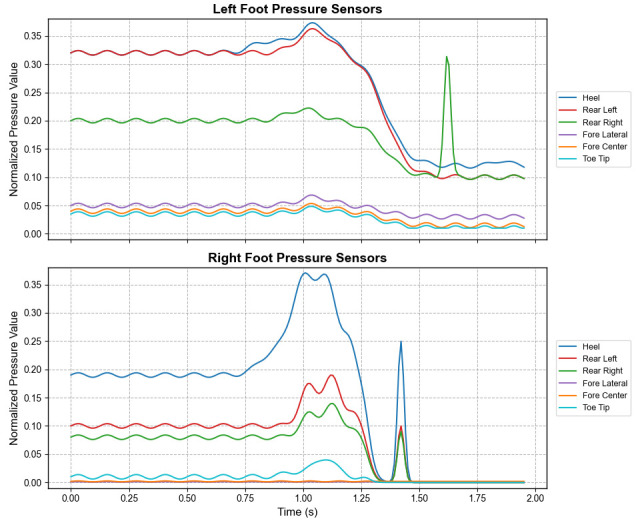
Plantar pressure variations in left and right feet during the stand-to-sit transition.

**Figure 9:**
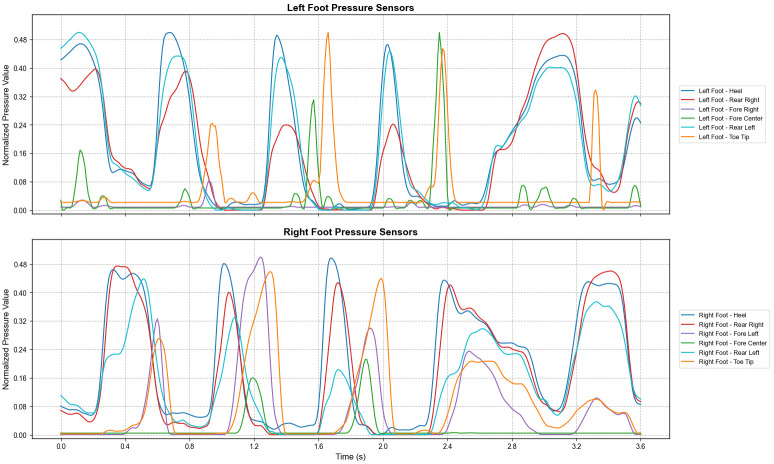
Periodic plantar pressure patterns of left and right feet during level walking.

As shown in Figure 8, during the stand-to-sit process, the heel pressure increases first while the forefoot pressure decreases rapidly, indicating a backward transfer of the COG. After sitting down, the pressure on both the heel and forefoot is lower than that in the standing state. Following the analysis of transitional movements, we also evaluated dynamic gaits; the periodic plantar pressure patterns of the left and right feet during level walking are illustrated in Figure 9.

The figure clearly shows a distinct walking periodicity, with the cycle duration for the left and right feet being similar (approximately 1.2–1.3 s) and appearing alternatingly.

The human gait cycle can be broadly divided into the Stance Phase and the Swing Phase. The Stance Phase accounts for approximately 60% of the cycle, representing the period when the foot is in contact with the ground, starting from heel strike and ending at toe-off. The Swing Phase accounts for approximately 40%, representing the period when the foot completely leaves the ground and swings forward [22]. Based on the characteristics of the piezoresistive sensors, the plantar pressure approaches zero during the Swing Phase, allowing for a clear distinction between the Stance and Swing Phases. This study introduces the concept of the “Dual Support Period” in gait analysis and subsequent algorithms, which refers to the interval within the gait cycle when both feet are in contact with the ground simultaneously. Ideally, the simultaneous lift-off of both feet (i.e., the disappearance of the Dual Support Period) is a critical criterion for distinguishing running from walking.

#### 3.1.2. Inertial Data Analysis

**Figure 10:**
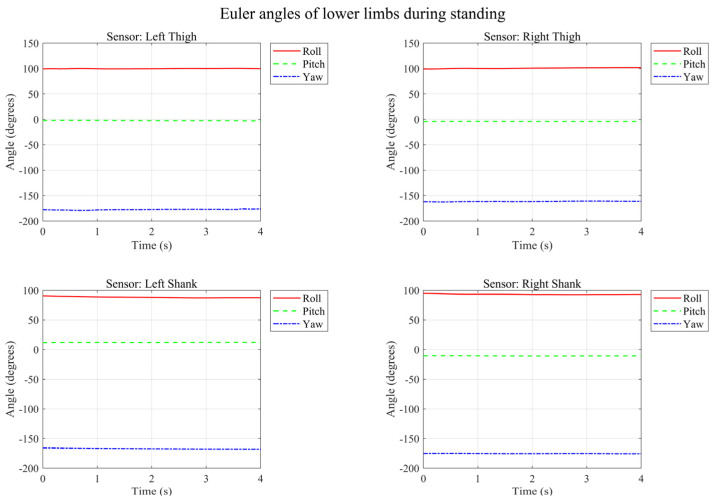
Temporal variation in lower limb Euler angles during standing.

By fusing the nine-axis data and solving for Euler angles based on quaternions, we first visualize the collected inertial data for static standing, as illustrated in Figure 10.

**Figure 11:**
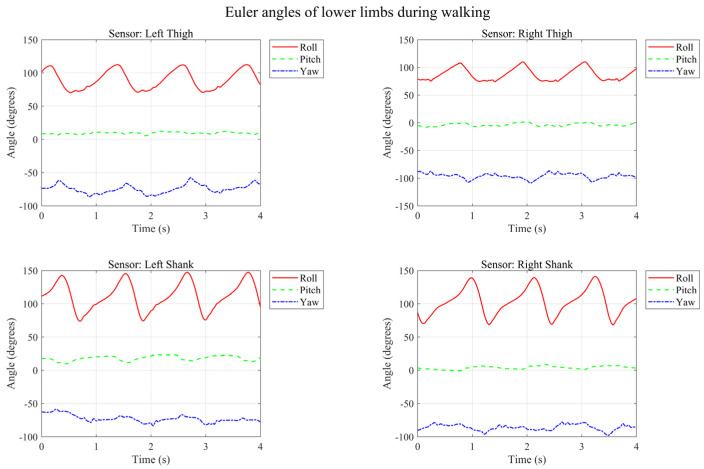
Temporal variation in lower limb Euler angles during level walking.

Data from static standing indicates that the drift issue of the IMU has been effectively mitigated through nine-axis data fusion: the accelerometer is accurate statically, while the gyroscope is precise dynamically. The two complement each other to minimize their respective defects, and the magnetometer provides an absolute directional reference to prevent long-term gyroscope drift. The temporal variation of lower limb Euler angles during level walking is illustrated in Figure 11.

From the time axis, it can be seen that one gait cycle is completed approximately every 1 s, which is consistent with normal walking speed. The left and right legs exhibit an alternating movement pattern, conforming to normal gait laws.

Biomechanically, the femur (thigh bone), as the longest bone in the human body, primarily bears weight and provides propulsion, mainly performing flexion and extension around the hip joint with a relatively simple trajectory. The tibia and fibula (shank bones) are relatively shorter and are primarily responsible for fine foot control and balance regulation.

**Figure 12:**
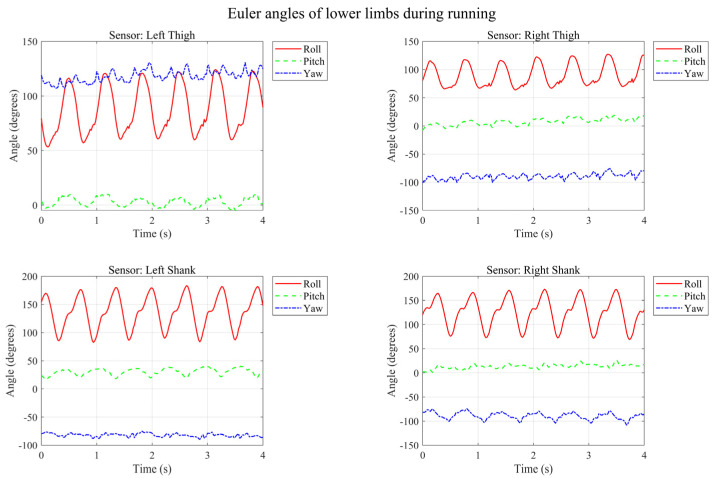
Temporal variation in Euler angles of lower limbs during running.

It can be observed that the Roll angle changes in the left and right thighs exhibit a phase difference of approximately 180 degrees. When the left leg is in the Stance Phase, the right leg is in the Swing Phase, which aligns with normal gait patterns. A brief transitional period can be observed in the Roll angle plot, corresponding to the Dual Support Period, which accounts for about 10–20% of the entire gait cycle, consistent with the characteristics of normal walking. The corresponding temporal variations of the lower limb Euler angles during running are shown in Figure 12.

**Figure 13:**
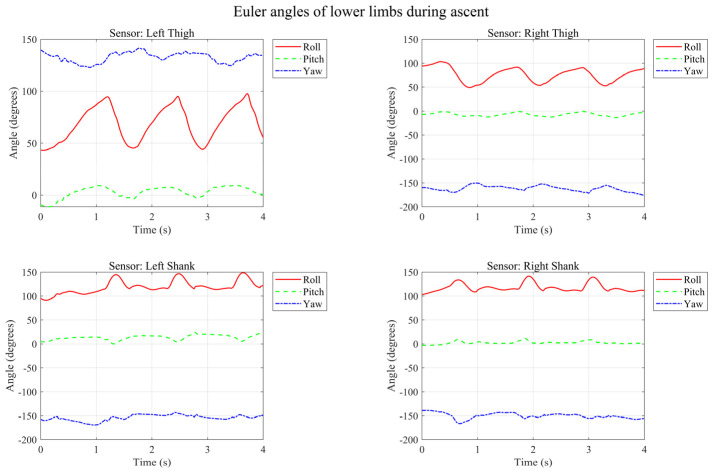
Temporal variation in Euler angles of lower limbs during ascent.

As seen in the running data, the Stance Phase is significantly shortened and the Dual Support Period disappears—this is the core characteristic of running. The cadence is approximately 2–2.5 steps per second (i.e., 120–150 steps/min). During stair ascent, as depicted in Figure 13, different kinematic characteristics emerge.

**Figure 14:**
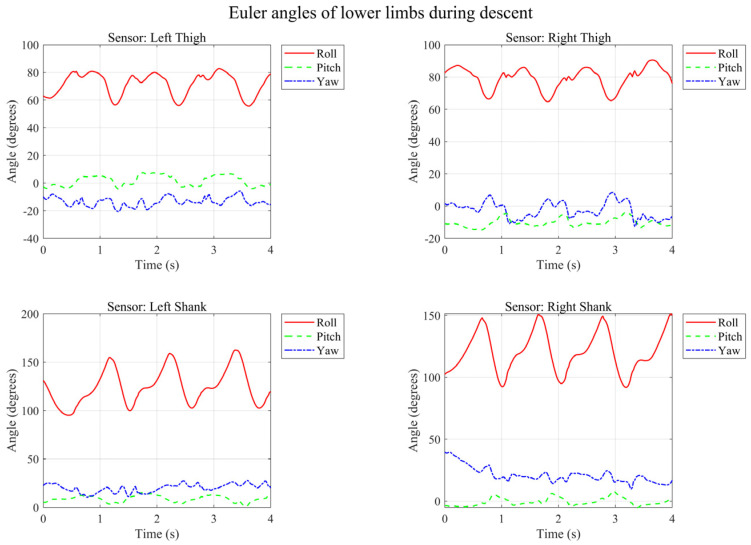
Temporal variation in Euler angles of lower limbs during descent.

During the ascent, the step frequency increases. As evidenced by the reduced and irregular Roll angle amplitude, the step length decreases, necessitating more time for balance adjustment. Compared to level walking, the fluctuations in the Pitch angle are more pronounced. Conversely, the Euler angle variations during stair descent are illustrated in Figure 14.

During stair ascent (going upstairs), the cadence increases. The decrease and irregular variation in the Roll angle amplitude indicate a reduced stride length and the need for time to adjust balance. The variation in the Pitch angle is more intense compared to level walking.

Based on the above analysis, the four motion modes exhibit distinct characteristics in terms of thigh/shank Roll amplitude, Pitch activity, and Yaw stability. The summary is presented in Table 2 below.

### 3.2. Overall Classification Performance

To evaluate the generalization capability of the proposed multi-modal fusion framework across different individuals, the Leave-One-Out Cross-Validation (LOOCV) strategy was adopted. Specifically, six rounds of experiments were conducted; in each round, data from one subject was selected as the test set, while the remaining five served as the training set.

Table 3 presents the detailed per-subject classification accuracies derived from the LOOCV. The system achieved a mean overall accuracy of 96.17%, with a low Standard Deviation (SD) of 2.48%. Furthermore, the 95% confidence interval (CI) was calculated to be [93.57%, 98.77%]. This relatively small Standard Deviation and tight confidence interval inherently demonstrate that the model maintains stable, high-precision performance, effectively accommodating variations in body types (e.g., height and weight) and individual walking habits without severe overfitting.

**Figure 15:**
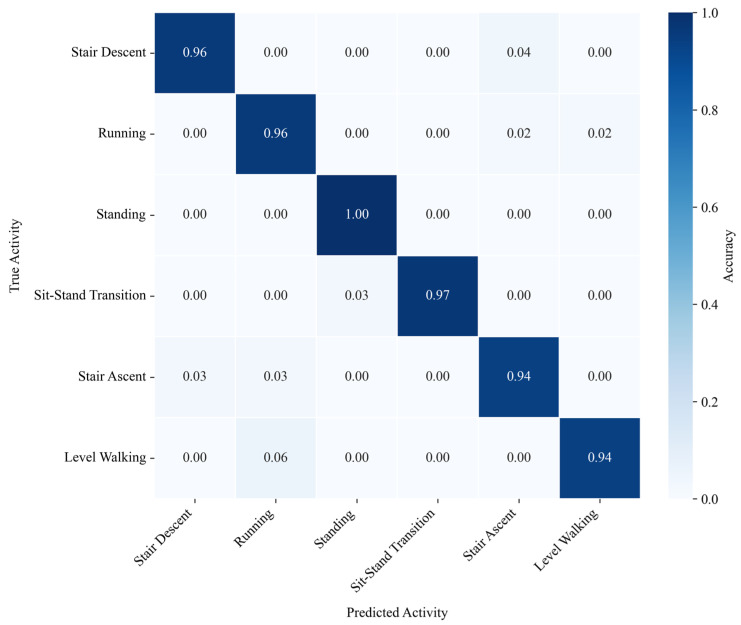
Confusion matrix of the gait recognition results under Leave-One-Out Cross-Validation.

Based on the aggregated results, the corresponding confusion matrix is shown in Figure 15.

Standing: Achieved the highest recognition accuracy of 100%. This demonstrates the superiority of the strategy that logically decouples dynamic and static gaits, effectively avoiding the interference of zero-bias drift from inertial sensors during static states.

- Sit–Stand Transitions: The recognition rate reached 97%. The system accurately captured the Center of Gravity (COG) transfer process and eliminated physically impossible state mutations.
- Running vs. Walking: The introduction of Dual Support Period detection significantly enhanced the distinction between running and walking.
- Stair Ascent/Descent: Some confusion occurred at the turning points or the start/end phases of the stairs, as the limb swing amplitudes (Roll/Pitch angles) were relatively similar to level walking during these specific phases.

### 3.3. Ablation Study on Sensor Modalities

**Figure 16:**
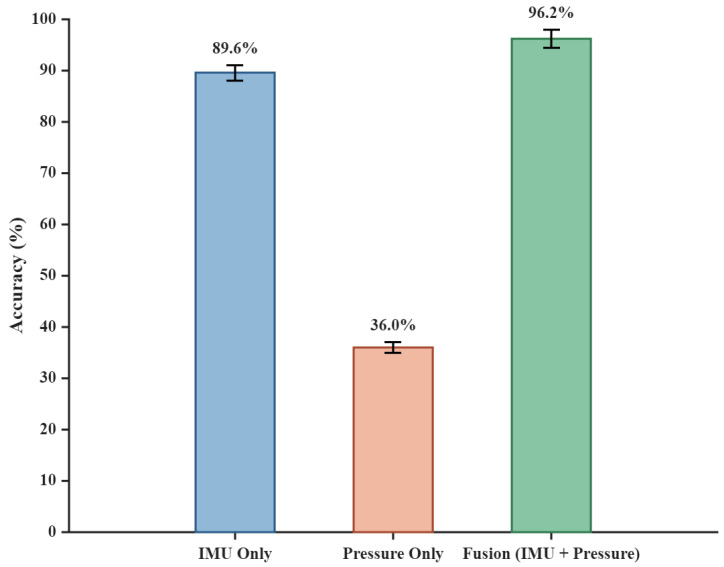
Comparison of overall gait recognition accuracy using single sensors versus the multi-modal fusion strategy with error bars indicating Standard Deviation.

To explicitly demonstrate the distinct contributions of each sensor modality and rigorously validate the necessity of the multi-modal fusion framework, a quantitative ablation study was conducted. Three sensing configurations were evaluated using the identical dataset, feature extraction pipeline, and Leave-One-Out Cross-Validation (LOOCV) strategy: IMU-only, plantar pressure-only, and the proposed fusion approach (IMU + pressure), with the comparative results presented in Figure 16.

The results demonstrate clear performance disparities among the configurations. The pressure-only model achieves an overall accuracy of just 36.0%, as it lacks spatial kinematic data during the Swing Phase, limiting its ability to distinguish complex dynamic gaits. Conversely, the IMU-only configuration reaches 89.6% by providing continuous spatial orientation. However, it struggles with static and transitional states (e.g., sitting versus standing) due to the absence of direct weight-bearing evidence.

By integrating both modalities, the proposed fusion approach achieves an optimal accuracy of 96.17%. This confirms that combining the reliable weight-bearing context of pressure sensors with the continuous kinematics of IMUs effectively leverages their complementary strengths, significantly enhancing the system’s robustness and precision across diverse activities.

### 3.4. Impact of Dimensionality Reduction

The handcrafted feature space constructed for the machine learning branch comprised 510 time-domain, frequency-domain, and biomechanical dimensions. Considering the heterogeneous and highly nonlinear nature of these multi-modal features, we strategically relied on the embedded feature selection mechanisms inherent to tree-based ensemble models, such as Random Forest and GBDT, rather than applying an unsupervised dimensionality reduction step. These models naturally evaluate feature importance based on criteria such as information gain during the node-splitting process, enabling dynamic, supervised feature prioritization.

To quantitatively validate this design choice, an additional ablation experiment was conducted using Principal Component Analysis (PCA) on the 510-dimensional feature space. Principal components explaining 95% of the total variance were retained, successfully reducing the dimensionality to 34. However, experimental results demonstrated a significant performance degradation following this explicit reduction. While the recognition of static postures remained stable, the accuracy for complex dynamic gaits dropped noticeably: stair ascent decreased from 94% to 78% and level walking dropped from 94% to 85%, culminating in a reduced overall accuracy of approximately 86%.

This degradation indicates that PCA, as an unsupervised linear transformation, inadvertently discards low-variance yet highly discriminative nonlinear spatiotemporal features (e.g., subtle Pitch angle variations between walking and stair climbing). In contrast, the proposed ensemble learning framework effectively preserves and utilizes these critical nonlinear features, justifying its robustness in handling high-dimensional gait data.

## 4. Discussion

### 4.1. Research Summary

This study addressed the limitations of existing gait recognition technologies, such as incomplete information from single-modal sensors, misjudgment of static actions due to drift, and difficulty in recognizing transition states. We proposed and validated a wearable gait recognition system based on multi-modal fusion.

In terms of hardware, we independently developed a low-power wearable device integrating Micro-Electro-Mechanical Systems (MEMS) Inertial Measurement Units (IMUs) and flexible-array pressure sensors. The system comprised four inertial sensing nodes and two plantar pressure sensing nodes (12 channels in total). Utilizing an ESP32 microcontroller as the core unit, the system achieved synchronized multi-source data acquisition at a sampling rate of 100 Hz and stable wireless Wi-Fi transmission.

In terms of algorithms, this study constructed a “coarse-to-fine” two-stage hierarchical recognition framework based on sensor characteristics.

- In the primary stage, utilizing the Time-Domain Energy and Frequency-Domain Significance of plantar pressure signals, a dual-threshold mechanism efficiently decouples static postures from dynamic gaits. This effectively solves the misjudgment problem caused by zero-bias drift in traditional inertial algorithms during standstill and reduces the computational burden.
- In the advanced classification stage, for static and transitional actions, a lightweight SVM model combined with a Finite State Machine (FSM) is employed to ensure the physical continuity of action logic. For complex dynamic gaits (walking, running, stairs), an ensemble learning model based on a Soft Voting mechanism is proposed. Based on a 1.28 s sliding window, the algorithm constructs a high-dimensional feature vector (totaling 510 dimensions, including 222 pressure features and 288 IMU features). This model fuses a Dual-Branch Neural Network (extracting deep features) with Random Forest and GBDT (utilizing structured time-frequency features) and introduces biomechanical indicators such as Dual Support Period, Center of Pressure (COP), and Range of Motion (ROM).

Experimental results demonstrate that under Leave-One-Out Cross-Validation (LOOCV), the system achieved a comprehensive recognition accuracy of 96.17% for eight types of daily activities. Specifically, the recognition accuracy for static postures (standing) reached 100%, transitional actions reached 97%, and complex dynamic gaits maintained an average accuracy above 94%. These results were validated through six rounds of Leave-One-Out Cross-Validation (LOOCV), demonstrating strong robustness. Furthermore, the introduction of Dual Support Period detection significantly improved the distinction between running and walking. This study validates the significant advantages of multi-modal data fusion in improving the accuracy of gait recognition, providing a reliable technical solution for applications such as rehabilitation monitoring, exoskeleton control, and human–machine interaction.

### 4.2. Future Research Direction

Future work will focus on the following three aspects:

**1. Edge Computing and Real-time Processing**

Although current data processing is executed on a host computer, the proposed hierarchical architecture is inherently optimized for embedded environments. Future work will focus on achieving full on-chip inference using TinyML techniques. The feasibility for this edge deployment is supported by three aspects:

Computational Complexity: The conditional execution strategy restricts the computationally intensive ensemble model exclusively to dynamic gaits, utilizing a lightweight SVM for static/transitional states. This substantially minimizes overall operations compared to continuous deep learning inference.

Memory Footprint: Processing the 1.28 s sliding window demands minimal dynamic memory, fitting easily within the standard 520 KB SRAM. Furthermore, applying INT8 quantization and tree pruning will compress the model’s Flash footprint to under 500 KB, well within the 4 MB limit.

Inference Latency: Bypassing deep learning components during static states ensures millisecond-level predictions. Achieving full on-device processing will eliminate wireless transmission delays and enhance user privacy, facilitating real-world applications in exoskeletons and rehabilitation.

**2. In-depth Biomechanical Analysis of Stair Climbing**

This study can distinguish stair climbing from level walking with high precision based on kinematic features. However, the biomechanical mechanisms during stair climbing require further investigation. Future research will combine current kinematic data (IMU) with kinetic data (pressure distribution) to estimate joint torques and muscle forces during stair locomotion, further improving recognition performance.

**3. Adaptation to Complex Real-world Environments**

Current experiments were conducted under controlled conditions. However, real-world scenarios involve complex movements, such as turning, sudden stops, and walking on uneven terrain (e.g., grass and slopes). Future work will focus on recognizing transitional gait events (e.g., turning while walking) and adapting the model to complex environments. We also plan to introduce more functions, such as estimating the turning radius using magnetometers and estimating terrain conditions using foot impact signals.

## Figures and Tables

**Table 1:** Definitions of the collected activities categorized by gait type.

Gait Category	Label	Action Name	Definition and Constraints
Static and Transitional (Processed by Static Branch)	1	Standing	Subject stands still in a natural posture with arms at sides.
	2	Sitting	Subject sits on a standard-height chair with feet flat on the ground.
	3	Sit-to-Stand	Subject rises from a sitting position to a fully standing state.
	4	Stand-to-Sit	Subject lowers from a standing state to a fully sitting position.
Dynamic Gaits (Processed by Dynamic Branch)	5	Walking	Level walking in a straight line at a uniform speed (~5 km/h).
	6	Running	Running in a straight line at a uniform speed (~10 km/h).
	7	Stair Ascent	Climbing stairs (step height ~15 cm) at a steady pace (~20 steps/min).
	8	Stair Descent	Descending stairs (step height ~15 cm) at a steady pace (~25 steps/min).

**Table 2:** Feature summary of different gait activities.

Gait Type	Level Walking	Running	Stair Ascent	Stair Descent
Cadence	1.0 steps/s	>2.0 steps/s	1.2–1.5 steps/s	0.75 steps/s
Thigh Roll Amplitude	100°	100°	60°	30°
Shank Roll Amplitude	100°	100°	70°	70°
Pitch Activity	Low	Low	High	Lowest
Yaw Stability	Stable	Stable	Unstable	Stable after transition
Locomotion Strategy	Efficiency-oriented	Speed-oriented	Power-oriented	Safety-oriented

**Table 3:** Per-subject classification accuracy and statistical metrics under LOOCV.

Subject	Accuracy (%)
A	92.16
B	94.44
C	98.52
D	97.40
E	98.30
F	96.20

## Data Availability

The raw data supporting the conclusions of this article will be made available by the authors on request. The data are not publicly available due to privacy and ethical restrictions regarding human subjects.

## References

[1] Sethi D., Bharti S., Prakash C. (2022). A comprehensive survey on gait analysis: History, parameters, approaches, pose estimation, and future work. Artif. Intell. Med..

[2] Xie J., Zhao H., Cao J., Qu Q., Cao H., Liao W.-H., Lei Y., Guo L. (2023). Wearable multisource quantitative gait analysis of Parkinson’s diseases. Comput. Biol. Med..

[3] Yashas B.Y., Santhosh L., Asha K.N., Potdar V., Shamanth R., Teja G.K. Gait Based Behaviometric Identification Using CASIA-B Dataset and Gait Energy Images. Proceedings of the 3rd IEEE International Conference on Knowledge Engineering and Communication Systems (ICKECS 2025).

[4] Qian G., Zhang J., Kidané A. (2008). People Identification Using Gait via Floor Pressure Sensing and Analysis. Lecture Notes in Computer Science (Including Subseries Lecture Notes in Artificial Intelligence and Lecture Notes in Bioinformatics).

[5] Chen C., Liu D., Wang X., Wang C., Wu X. An Adaptive Gait Learning Strategy for Lower Limb Exoskeleton Robot. Proceedings of the 2017 IEEE International Conference on Real-Time Computing and Robotics (RCAR).

[6] Chen Y., Xue Y. A Deep Learning Approach to Human Activity Recognition Based on Single Accelerometer. Proceedings of the 2015 IEEE International Conference on Systems, Man, and Cybernetics.

[7] Zebin T., Scully P.J., Ozanyan K.B. Human Activity Recognition with Inertial Sensors Using a Deep Learning Approach. Proceedings of the IEEE Sensors Conference (ICSENS 2016).

[8] Bayat A., Pomplun M., Tran D.A. (2014). A Study on Human Activity Recognition Using Accelerometer Data from Smartphones. Procedia Comput. Sci..

[9] Liu Y., Liu Y., Song Q., Wu D., Jin D. (2024). Gait Event Detection Based on Fuzzy Logic Model by Using IMU Signals of Lower Limbs. IEEE Sens. J..

[10] Xia C., Sugiura Y. Wearable Accelerometer Optimal Positions for Human Motion Recognition. Proceedings of the 2020 IEEE 2nd Global Conference on Life Sciences and Technologies (LifeTech).

[11] Andersson R., Bermejo-García J., Agujetas R., Cronhjort M., Chilo J. (2024). Smartphone IMU Sensors for Human Identification through Hip Joint Angle Analysis. Sensors.

[12] Muro-de-la-Herran A., Garcia-Zapirain B., Mendez-Zorrilla A. (2014). Gait Analysis Methods: An Overview of Wearable and Non-Wearable Systems, Highlighting Clinical Applications. Sensors.

[13] Wang J., Dai Y., Kang T., Si X. Research on Gait Recognition Based on Lower Limb EMG Signal. Proceedings of the 2021 IEEE International Conference on Mechatronics and Automation (ICMA).

[14] LaMont J.G. (1986). Functional Anatomy of the Lower Limb. Clin. Plast. Surg..

[15] Chen B., Wang X., Huang Y., Wei K., Wang Q. (2015). A Foot-Wearable Interface for Locomotion Mode Recognition Based on Discrete Contact Force Distribution. Mechatronics.

[16] Wang Q., Guan H., Wang C., Lei P., Sheng H., Bi H., Hu J., Guo C., Mao Y., Yuan J. (2025). A Wireless, Self-Powered Smart Insole for Gait Monitoring and Recognition via Nonlinear Synergistic Pressure Sensing. Sci. Adv..

[17] Cha Y., Song K., Shin J., Kim D. Gait Analysis System Based on Slippers with Flexible Piezoelectric Sensors. Proceedings of the 2018 IEEE International Conference on Robotics and Biomimetics (ROBIO).

[18] Young A.J., Hargrove L.J. (2016). A Classification Method for User-Independent Intent Recognition for Transfemoral Amputees Using Powered Lower Limb Prostheses. IEEE Trans. Neural Syst. Rehabil. Eng..

[19] Yunas S.U., Alharthi A., Ozanyan K.B. Multi-Modality Sensor Fusion for Gait Classification Using Deep Learning. Proceedings of the 2020 IEEE Sensors Applications Symposium (SAS).

[20] Willems T.M., De Ridder R., Roosen P. (2012). The effect of a long-distance run on plantar pressure distribution during running. Gait Posture.

[21] Liu Z., Yin Z., Jiang Y., Zheng Q. (2022). Dielectric interface passivation of polyelectrolyte-gated organic field-effect transistors for ultrasensitive low-voltage pressure sensors in wearable applications. Mater. Today Electron..

[22] Han Y.C., Wong K.I., Murray I. (2019). Gait Phase Detection for Normal and Abnormal Gaits Using IMU. IEEE Sens. J..

